# A perfectly stoichiometric and flat CeO_2_(111) surface on a bulk-like ceria film

**DOI:** 10.1038/srep21165

**Published:** 2016-02-16

**Authors:** C. Barth, C. Laffon, R. Olbrich, A. Ranguis, Ph. Parent, M. Reichling

**Affiliations:** 1 Aix-Marseille University, CNRS, CINaM UMR 7325, 13288 Marseille, France; 2 Fachbereich Physik, Universität Osnabrück, Barbarastr. 7, 49076 Osnabrück, Germany

## Abstract

In surface science and model catalysis, cerium oxide (ceria) is mostly grown as an ultra-thin film on a metal substrate in the ultra-high vacuum to understand fundamental mechanisms involved in diverse surface chemistry processes. However, such ultra-thin films do not have the contribution of a bulk ceria underneath, which is currently discussed to have a high impact on in particular surface redox processes. Here, we present a fully oxidized ceria thick film (180 nm) with a perfectly stoichiometric CeO_2_(111) surface exhibiting exceptionally large, atomically flat terraces. The film is well-suited for ceria model studies as well as a perfect substitute for CeO_2_ bulk material.

Cerium oxide (ceria) is a most important material in heterogeneous catalysis^1^ due to its high oxygen storage capacity (OSC) based on the oxidation and reduction of cerium ions^2^. It is used in the water-gas-shift reaction and for the oxidation of hydrocarbons^2^, with the most prominent application being the three-way-catalyst^3^,^4^.

To understand redox processes and related surface chemistry^5^ at the atomic scale, ultra-thin ceria films have been used as model systems in surface science and heterogeneous model catalysis. In particular, the CeO_2_(111)^6^,^7^,^8^ and its reduced variant Ce_2_O_3_(111)^9^,^10^ have been studied, and both reduction as well as re-oxidation have been demonstrated^10^,^11^,^12^,^13^,^14^. However, such studies are not suitable for understanding surface processes on thick ceria material as oxygen diffusion from the bulk may strongly influence the surface reaction state^2^. Applications of ceria in catalysis, sensor technology as well as ceria catalysis model studies require materials with a well-defined surface and oxygen storage capacity.

Recently, a method for the growth of 150 to 250 nm thick fully oxidized ceria films on Si(111) by molecular beam epitaxy (MBE) has been proposed^15^,^16^, and such films were suggested as an easy-to-prepare material to replace bulk ceria crystals^15^,^17^,^18^ that are most difficult to grow^19^. We show that a straightforward three step procedure in ultra-high vacuum (UHV) following MBE growth of the film yields a fully oxidized CeO_2_(111) surface with exceptionally wide, clean and atomically flat terraces. This is substantiated by UHV noncontact atomic force microscopy (NC-AFM) and photoelectron spectroscopy (XPS) measurements. Our work demonstrates that even ceria surfaces that are exposed to the ambient air and transferred into the UHV can be cleaned and oxidized by this three step procedure.

## Results

**Figure 1:**
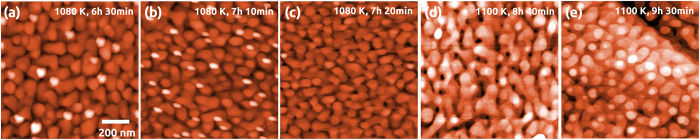
Development of the surface morphology of a ceria film subjected to consecutive annealing cycles performed at 1080 (**a**–**c**) and 1100 K (**d**,**e**) as determined by tapping mode AFM measurements in air. The specified times denote the total time of the heating part of each annealing cycle (see Fig. S1 in the Supplemental Material), whereas the specified maximum temperature is kept for at least 1 h. Image size of all images: 1.0 × 1.0 *μ*m^2^, color coded height scale: 14.0 nm (**a**–**c**), 5.0 nm (**d**) and 6.3 nm (**e**) correspond to white color.

In previous work, we found that annealing the ceria film in UHV at temperatures above 1050 K yields the high-temperature surface morphology characterized by large atomically rough terraces^18^ and a strong (near-)surface reduction^17^. Step-wise annealing the sample in air yields similar results as demonstrated by the series of AFM images shown in Fig. 1, representing five heating and cooling cycles performed on one sample with maximum temperatures of 1080 and 1100 K, respectively. For the first two annealing steps performed at 1080 K (Fig. 1(a)), remainders of pyramids dominating the low-temperature surface morphology are found as for films annealed in UHV^18^. After the fourth and in particular the fifth annealing cycle where the temperature is increased to 1100 K to accelerate the surface transformation, the surface is considerably flattened exhibiting mainly layered islands, some of them approaching a hexagonal shape (Fig. 1(d,e)) like on the surface of bulk crystals^20^.

**Figure 2:**
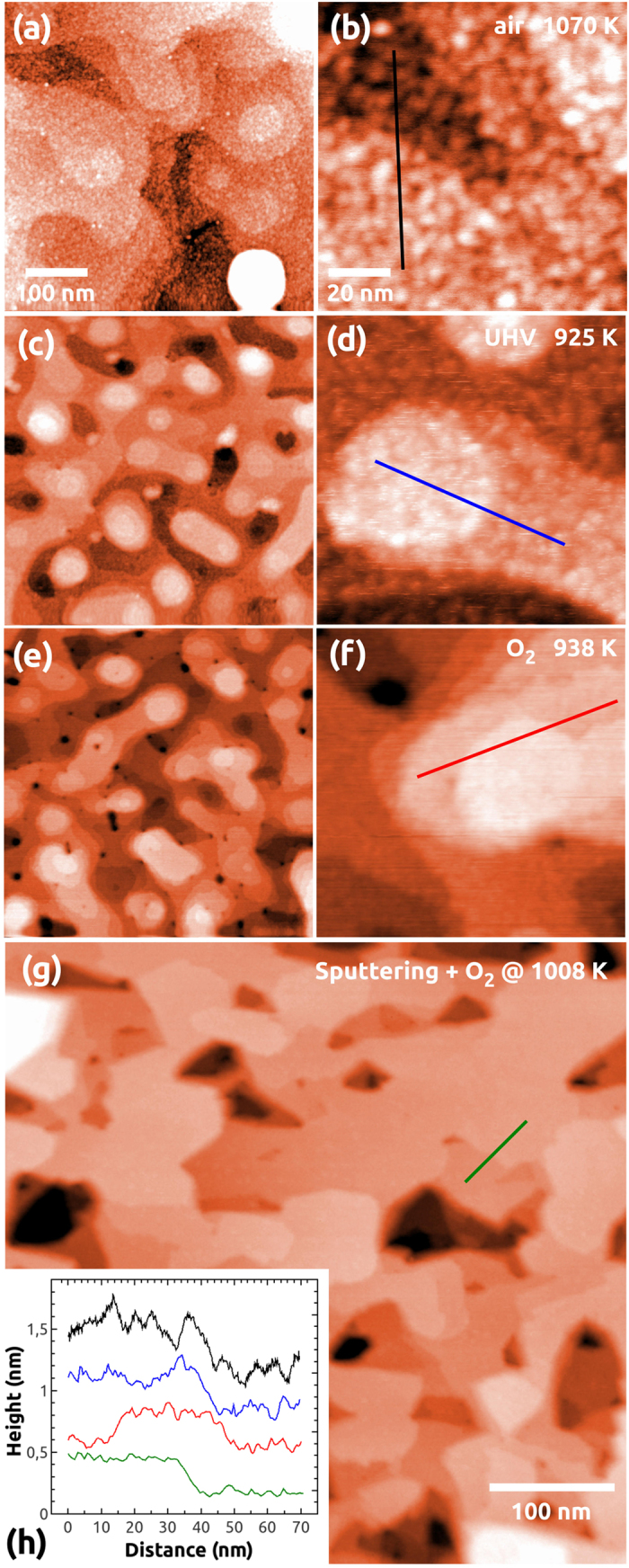
NC-AFM topography images obtained in UHV on a film annealed in air. (**a**,**b**) Surface after air-annealing. (**c**,**d**) Surface after annealing in UHV at 925 K for 1 h. (**e**,**f**) Surface after annealing in oxygen 
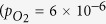
 mbar) at 938 K for 1 h. (**g**) Surface after Ar^+^ bombardment 
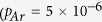
 mbar, 1.5 keV, 5 min) and annealing in oxygen 
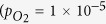
 mbar) at 1008 K for 1 h. Image sizes: 500 × 500 nm^2^ (**a**,**c**,**e**,**g**), 100 × 100 nm^2^ (**b**,**d**,**f**), color coded height scale: 1.5 nm (**a**), 1.2 nm (**b**), 1.5 nm (**c**), 1.5 nm (**d**), 3.1 nm (**e**), 3.1 nm (**f**) and 9.4 nm (**g**). (**h**) Height profiles taken along the lines in images (**b**,**d**,**f**,**g**). Line colors in NC-AFM images correspond to line colors of the profiles.

In the images shown in Fig. 1, terraces appear as rather flat and intersected by steps with a height of integer multiples of the 0.315 nm triple-layer step height. However, when imaging the air-annealed film by NC-AFM in UHV, the terraces do not appear atomically flat, as it can be seen in the topography image of Fig. 2(a,b): apart from the step and terrace structure, a granular structure formed by small features with a height below 0.3 nm covers the terraces (see black profile in Fig. 2(h)). This is similar to granular structures observed on other dielectric crystals like CaF_2_(111)^21^ and MgO(001)^22^, in particular after air exposure.

**Figure 3:**
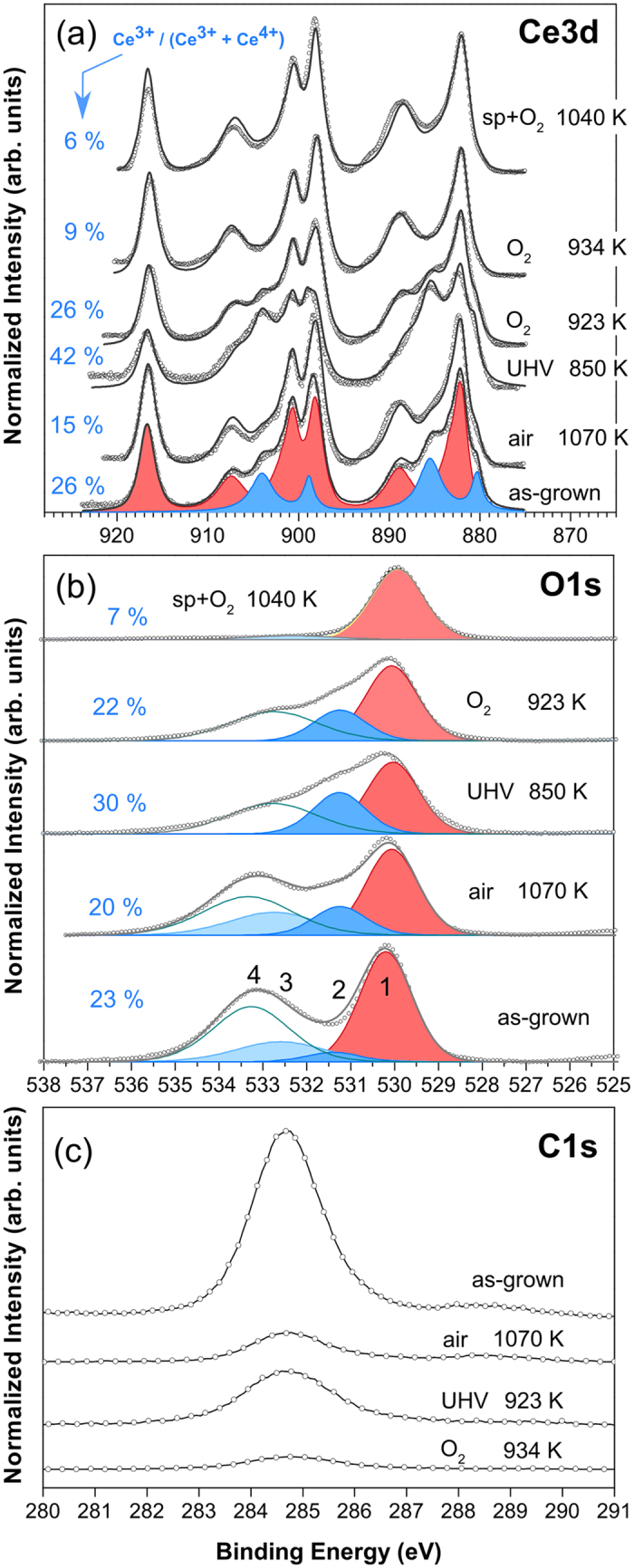
XPS Ce3d (**a**), O1s (**b**) and C1s (**c**) spectra taken on a ceria film in different states of preparation. The sequence of preparation steps is: as-grown, annealing in air, annealing in UHV (923 K, 1 h), two times annealing in oxygen 
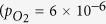
 mbar, 934 and 938 K, each 1 h) and sputtering 
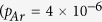
 mbar, 1.0 keV, 15 min) followed by annealing in oxygen 
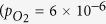
 mbar, 1040 K, 1 h). Circles denote measured points while the solid lines are fit curves. The Ce^3+^ and O-Ce^3+^ peaks are in blue, whereas the Ce^4+^ and O-Ce^4+^ peaks are in red. The assignment of peaks 1 to 4 in frame (**b**) is given in the main text.

To reveal the oxidation state of the ceria film and surface contaminants, we analyze the Ce3d, O1s and C1s peaks of XPS spectra taken after different preparation steps as shown in Fig. 3. By analyzing survey spectra (not shown), we make sure not to miss any contamination. Details of the quantitative spectral analysis by fitting model curves to spectral features are given in the Supplementary Information. To determine the relative content of Ce^3+^ in the film, we first analyze the Ce3d spectra by relating the area of the Ce^3+^ doublet 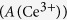 to the total multiplet area 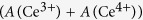 and determine the reduction state in % by calculating the fraction 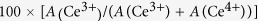
10 as shown in Fig. 3(a). The uncertainties on the fraction is estimated to be around ±5% (see Supplementary Information).

Next, we analyze the O1s spectra shown in Fig. 3(b) by fitting the spectrum with 4 Gaussian functions at 530.2 eV (peak 1), 531.4 eV (peak 2), 532.6 eV (peak 3) and 533.3 eV (peak 4). Peak 1 and 2 are assigned to oxygen bound to Ce^4+^ and Ce^3+^, respectively^23^,^24^ whereas peak 3 is assigned to hydroxyls and/or H_2_O molecules bound to Ce^3+^ 25,26. From Peaks 1 and 2, we extract the relative content of Ce^3+^. Note, however, that signals for the O1s peak originate from significantly deeper layers (~1.5 nm) than signals from the Ce3d peak (~1.0 nm) explaining different values for the reduction state^27^. Peak 4 remains constant during all thermal treatment steps and is associated to SiO_2_^28^ originating from oxidized silicon particles contaminating the film during cutting the Si wafer. This interpretation is supported by the observation that a 2s and 2p peak of SiO_2_ at 106 eV and 156 eV, respectively, are observed in related survey spectra (not shown). A segregation of Si from the substrate to the surface can, however, be excluded as this takes place only at temperatures high enough to destroy the film^29^.

From the Ce3d spectrum it can be concluded that the Ce^3+^ concentration of the as-grown film is 26% and decreases to 15% during air annealing indicating that the surface of the film is significantly oxidized. This trend of oxidation is weaker in deeper layers since the O-Ce^3+^ peak becomes only slightly smaller in the O1s spectra (from 23 to 20%). The hydroxyl peak in the O1s spectrum is, however, not affected by annealing in air. As it can be seen in the C1s spectrum (Fig. 3(c)), the as-grown film is, furthermore, contaminated with carbon containing species. However, the C1s peak decreases significantly upon annealing the film in air. We anticipate that oxygen from the air reacts off carbon containing species.

In an attempt to completely remove carbon and hydroxyl containing species, we anneal the sample in UHV for one hour at a temperature of 850 K in the XPS chamber and similarly at 925 K in the AFM chamber. After such annealing, the film is strongly reduced as the surface Ce^3+^ concentration in the Ce3d spectra increases to 42%, accompanied by a reduction in deeper layers (30%, O1s spectrum). Surface cleaning by this step is immediately evident from the O1s spectra. The hydroxyl peak 3 disappears completely as hydroxyls and water desorb at temperatures higher than 400 K^25^,^26^. However, in the highly resolved NC-AFM images of Fig. 2(c,d), the granular structure is still visible, although, reduced to some extent. Interestingly, the C1s XPS peak increases (Fig. 3(c)) what we attribute to residual carbon segregating from subsurface regions to the surface.

After the reduction of the ceria film by UHV annealing, we seek for a re-oxidation and anneal the film for one hour at 938 K (AFM) and 923 K (XPS), in molecular oxygen (
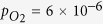
 mbar). The NC-AFM topography images in Fig. 2(e,f) clearly show, that the granular structure disappears and that the terraces are almost atomically flat. Surface cleaning is also reflected in the carbon C1s peak almost completely disappearing already after the first oxygen annealing step and being reduced to zero in the second annealing step. However, annealing in oxygen does not only clean the surface but also strongly oxidizes the film what is reflected in the Ce^3+^ concentration decreasing from 42% first to 26% and then to 9% in the second oxygen annealing step at 934 K.

Based on the XPS results, we assign the topographic features seen in Fig. 2(a,b) to carbon and hydroxyl containing species where the hydroxyl containing species can be removed by annealing in UHV while carbon contaminants visible in Fig. 2(c,d) can be only removed by annealing in molecular oxygen. Already after the first annealing in oxygen, an almost stoichiometric and almost atomically flat CeO_2_(111) surface as shown in Fig. 2(e,f) is obtained.

The only surface contaminant that cannot be removed by heat and oxygen treatment are the SiO_2_ particles. As samples are carefully rinsed with water and wiped after cutting the wafer, we anticipate that residual SiO_2_ particles are very small and fill some of the many pits left by surface preparation. To remove this contaminant, we sputter-clean the film with Ar^+^ ions and anneal again for one hour at 1008 K in molecular oxygen. After this final preparation step, we obtain a surface with atomically flat terraces having an extension of up to several 100 nm, intersected by one to three triple-layer high steps as seen in Fig. 2(g) and line profiles compiled in Fig. 2(h). The step edges form angles of 60 and 120 , as expected for the growth of a high-quality CeO_2_(111) surface. The analysis of XPS spectra reveals that the SiO_2_ contamination can effectively be removed as evident from the extremely small peak 4 in the respective O1s spectrum shown in Fig. 3(b). Another remarkable result is that the surface is now almost completely oxidized. Note, that the final values of 6% and 7% for the Ce^3+^ concentration determined from the analysis of the Ce3d and O1s peaks are very close to each other indicating that not only the surface but also near surface layers are finally well oxidized.

## Conclusions

In conclusion, a stoichiometric CeO_2_(111) surface with exceptionally wide atomically flat terraces and a reservoir of oxygen in near surface layers can be prepared on a thick ceria film by a combination of simple procedures comprising annealing in air, sputter-cleaning and annealing in UHV, and annealing in UHV back-filled with oxygen. The surface quality and purity of this system is better or at least equal compared to what can be expected from a well-prepared surface of a grown bulk crystal.

Whereas the sputtering-cleaning is a particular preparation step needed for the ceria-silicon system to remove residual SiO_2_ contaminants from the surface, the annealing in molecular oxygen is needed to fully oxidize the surface but also to remove contaminants like hydroxyls and carbon containing surface species if the film has been exposed before to the ambient air. With respect to the latter, we propose that any air-exposed ceria surface can be cleaned and fully oxidized by a high-temperature annealing in molecular oxygen such that ceria samples can be transported in air between two UHV systems.

In general, due to its simplicity of MBE growth, surface preparation and integration into silicon technology, the thick ceria film we present here is an ideal choice for surface science studies, catalysis research and applications where a well-prepared ceria surface with oxygen storage capacity is required.

## Methods

Ceria films with a thickness of 180 nm are grown in one batch with a 3 nm thick hexagonal Pr_2_O_3_(0001) buffer layer on Si(111) by MBE as described earlier^15^, cut from the wafer into pieces of 10 × 10 mm^2^ by an abrasive wire saw and then stored for several months under ambient laboratory conditions. To reveal the surface morphology and its change during preparation, tapping mode atomic force microscopy (XE 100 AFM, Park Scientific (Suwon, Korea) and Nanoscope Multimode III, Bruker (Billerica MA, USA)) is used for surface imaging under ambient conditions and NC-AFM is accomplished in UHV (RT-AFM/STM, Scienta Omicron GmbH (Taunusstein, Germany)). For studying the development of the surface stoichiometry, samples from the same batch are subjected to the same thermal treatment and analyzed by XPS in a second UHV system (un-monochromatized X-ray source, PSP Vacuum Technology (Macclesfield, UK)). The first preparation step is always annealing the sample in air with a furnace providing temperatures of up to 1100 K. For annealing in UHV, a furnace mounted in the NC-AFM vacuum chamber is used^22^ while heating in the XPS system is accomplished by electron bombardment of the back of the sample holder plate. Further technical details of sample preparation, AFM and NC-AFM measurements can be found in the Supplementary Information.

## Additional Information

**How to cite this article**: Barth, C. *et al.* A perfectly stoichiometric and flat CeO_2_(111) surface on a bulk-like ceria film. *Sci. Rep.* **6**, 21165; doi: 10.1038/srep21165 (2016).

## Supplementary Material

Supplementary Information

## References

[b1] Trovarelli A. & Fornasiero P. Catalysis by ceria and related materials. 2nd edn (Imperial College Press, London, 2013).

[b2] Gorte R. J. Ceria in catalysis: From automotive applications to the water-gas shift reaction. AIChE J. 56, 1126–1135 (2010).

[b3] Di Monte R. & Kašpar J. On the role of oxygen storage in three-way catalysis. Topics Catal. 28, 47–57 (2004).

[b4] Matsumoto S. Recent advances in automobile exhaust catalysts. Catal. Today 90, 183–190 (2004).

[b5] Paier J., Penschke Ch. & Sauer J. Oxygen defects and surface chemistry of ceria: quantum chemical studies compared to experiment. Chem. Rev. 113, 3949–85 (2013). 23651311 10.1021/cr3004949

[b6] Eck S., Castellarin-Cudia C., Surnev S., Ramsey M. G. & Netzer F. P. Growth and thermal properties of ultrathin cerium oxide layers on Rh (111). Surf. Sci. 520, 173–185 (2002).

[b7] Lu J.-L., Gao H.-J., Shaikhutdinov S. & Freund H.-J. Morphology and defect structure of the CeO_2_(111) films grown on Ru(0001) as studied by scanning tunneling microscopy. Surf. Sci. 600, 5004–5010 (2006).

[b8] Grinter D. C., Ithnin R., Pang Ch. L. & Thornton G. Defect structure of ultrathin ceria films on Pt(111): Atomic views from scanning tunnelling microscopy. J. Phys. Chem. C 114, 17036–17041 (2010).

[b9] Stetsovych V. *et al.* Epitaxial cubic Ce_2_O_3_ films via Ce–CeO_2_ interfacial reaction. J. Phys. Chem. Lett. 4, 866–871 (2013). 26291348 10.1021/jz400187j

[b10] Luches P., Pagliuca F. & Valeri S. Structural and morphological modifications of thermally reduced cerium oxide ultrathin epitaxial films on Pt(111). Phys. Chem. Chem. Phys. 16, 18848–18857 (2014). 25079214 10.1039/c4cp02723j

[b11] Nie J. C., Yamasaki H., Yamada H., Nakagawa Y. & Develos-Bagarinao K. Self-assembled CeO_2_ buffer layers on R-cut sapphire for high-current-density YBa_2_Cu_3_O_7−*δ*_ films. Supercond. Sci. Technol. 16, 768–772 (2003).

[b12] Nie J. C. & Yamasaki H. High density of nanodots on atomically flat CeO_2_ buffer layers for inducing effective vortex-pinning centers in YBa_2_ Cu_3_ O_7−*δ*_ films on sapphire. Thin Solid Films 515, 2577–2581 (2006).

[b13] Flege J.- I. *et al.* Ultrathin, epitaxial cerium dioxide on silicon. Appl. Phys. Lett. 104, 131604 (2014).

[b14] Allahgholi A., Flege J.-I., Thieß S., Drube W. & Falta J. Oxidation-state analysis of ceria by X-ray photoelectron spectroscopy. Chem. Phys. Chem. 16, 1083–1091 (2015). 25703923 10.1002/cphc.201402729

[b15] Zoellner M. H. *et al.* Stacking behavior of twin-free type-B oriented CeO_2_(111) films on hexagonal Pr_2_O_3_(0001)/Si(111) systems. Phys. Rev. B 85, 035302 (2012).

[b16] Niu G. *et al.* Controlling the physics and chemistry of binary and ternary praseodymium and cerium oxide systems. Phys. Chem. Chem. Phys. 17, 4513 (2015). 10.1039/c5cp02283e 26355535

[b17] Pieper H. H. *et al.* Morphology and nanostructure of CeO_2_(111) surfaces of single crystals and Si(111) supported ceria films. Phys. Chem. Chem. Phys. 14, 15361–15368 (2012). 23060225 10.1039/c2cp42733h

[b18] Olbrich R. *et al.* A well-structured metastable ceria surface. Appl. Phys. Lett. 104, 081910 (2014).

[b19] Tani E., Yoshimura M. & Sōmiya S. In Hydrothermal reactions for materials science and engineering - an overview of research in Japan (ed Sōmiya S.) 220–226 (Springer: Netherlands, London, , 1989).

[b20] Torbrügge S., Cranney M. & Reichling M. Morphology of step structures on CeO_2_(111). Appl. Phys. Lett. 93, 073112 (2008).

[b21] Reichling M., Huisinga M., Gogoll S. & Barth C. Degradation of the CaF_2_(111) surface by air exposure. Surf. Sci. 439, 181–190 (1999).

[b22] Barth C., Claeys C. & Henry C. R. Surface preparation of hard ionic crystals by ultrahigh vacuum cleavage. Rev. Sci. Instr. 76, 083907 (2005).

[b23] Mullins D. R., Overbury S. H. & Huntley D. R. Electron spectroscopy of single crystal and polycrystalline cerium oxide surfaces. Surf. Sci. 409, 307–319 (1998).

[b24] Hasegawa T. *et al.* Epitaxial growth of CeO_2_(111) film on Ru(0001): Scanning tunneling microscopy (STM) and x-ray photoemission spectroscopy (XPS) study. J. Chem. Phys. 140, 044711 (2014). 25669571 10.1063/1.4849595

[b25] Kundakovic Lj., Mullins D. R. & Overbury S. H. Adsorption and reaction of H_2_O and CO on oxidized and reduced Rh/CeO_*x*_(111) surfaces. Surf. Sci. 457, 51–62 (2000).

[b26] Henderson M. A., Perkins C. L., Engelhard M. H., Thevuthasan S. & Peden C. H. F. Redox properties of water on the oxidized and reduced surfaces of CeO_2_(111). Surf. Sci. 526, 1–18 (2003).

[b27] Krawczyk M., Holdynski M., Lisowski W., Sobczak J. W. & Jablonski A. Electron inelastic mean free paths in cerium dioxide. Appl. Surf. Sci. 341, 196–202 (2015).

[b28] Preisler E. J., Marsh O. J., Beach R. A. & McGill T. C. Stability of cerium oxide on silicon studied by x-ray photoelectron spectroscopy. J. Vac. Sci. Technol. B 19, 1611–1618 (2001).

[b29] Wilkens H. *et al.* Structural transitions of epitaxial ceria films on Si(111). Phys. Chem. Chem. Phys. 15, 18589–18599 (2013). 24076746 10.1039/c3cp52688g

