# Supplementary material for: A perfectly stoichiometric and flat CeO2(111) surface on a bulk-like ceria film
Source: Sci Rep. 2016 Feb 16;6:21165. doi: 10.1038/srep21165 (PMC4754737; doi:10.1038/srep21165)
Supplement: Supplementary Information [file srep21165-s1.pdf]

# – Supplementary Information –
A perfectly stoichiometric and flat $CeO_2(111)$ surface on a bulk-like ceria film

C. Barth,[1] C. Laffon,[1] R. Olbrich,[2] A. Ranguis,[1] Ph. Parent[1] and M. Reichling[2]
[1]*Aix-Marseille University, CNRS, CINaM UMR 7325, 13288 Marseille, France* and
[2]*Fachbereich Physik, Universität Osnabrück, Barbarastr. 7, 49076 Osnabrück, Germany*

SAMPLE PREPARATION

The ceria films are annealed in air in a cylindrical type T7 furnace based on the Chevenard-Joumier system (ADAMEL, Paris, France). The oven has two ends left open to guarantee a constant flow of fresh air during annealing. The sample being almost entirely enclosed by the heating oven walls ensures a homogeneous heating and a precise temperature measurement by a K-type thermocouple placed next to the sample. However, due to the air flow inside the furnace the surface temperature is lower than measured explaining that the temperatures reported here are slightly higher than the ones reported previously [1]. Due to the large thermal mass of the furnace, the target temperature is reached after about 10 hours (Fig. S1(a)), so that heating and cooling of the sample is always very slow. At a temperature of 700 K, the heating rate is below 3 K/min and decreases at higher temperatures to values between 2 to 0.5 K/min. The cooling rate is below 5 K/min and decreases afterwards.

After annealing in air, the sample is fixed by thin tantalum wires spot welded to a tantalum plate compatible with the sample transfer system of the UHV NC-AFM (Omicron NanoTechnology, Taunusstein, Germany). XPS and NC-AFM experiments are conducted in two separate ultra-high vacuum (UHV) chambers allowing for further annealing the samples either in UHV or in an oxygen atmosphere (10^{-6} to 2×10^{-5} mbar oxygen partial pressure). For annealing in the NC-AFM chamber, a home-made cylindrical furnace is used as described earlier [2]. The furnace heats the sample mainly by radiative heat transfer and a K-type thermocouple placed inside the furnace without direct contact to the sample is used for precise temperature measurements.

For XPS measurements, similarly prepared samples are transferred into the XPS chamber. For annealing, the sample is heated by a combined radiant and electron bombardment system (Meca 2000, Vinci-Technologies, Nanterre, France) in the XPS chamber. The temperature is measured with a pyrometer (IMPAC IGA 5 MB20, LumaSense Technologies, Santa Clara, USA) through a quartz UHV window.

Typical values for the speed of heating and cooling in the NC-AFM and XPS chambers are 10 to 20 K/min (Fig. S1(b)). For the oxidation experiments, each vacuum chamber is back-filled with molecular oxygen (NC-AFM: 99,995 % purity, Linde MINICAN, Munich, Germany, XPS: 99.998% purity, Air Liquide, Paris, France) via a leakage valve while constantly monitoring the pressure.

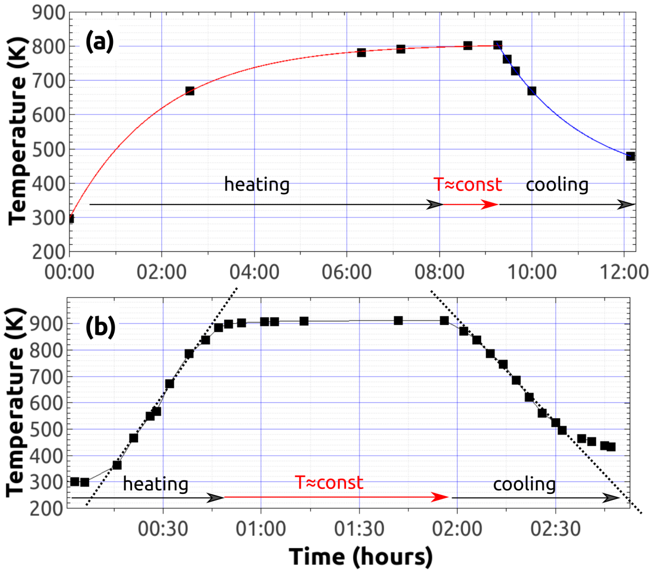

Figure S1. Typical temperature vs. time curves representing the heating and cooling parts during the annealing of the ceria films. (a) Annealing of the sample in air with a furnace with a heating rate below 3 K/min for $T \approx 700$ K and 0.5 to 2 K/min for $T > 700$ K. The cooling rate is below 5 K/min for $T \approx 800$ K. (b) Annealing of a sample in the UHV furnace of the NC-AFM chamber with heating and cooling rates of 18 and 11 K/min, respectively.

AFM AND NC-AFM MEASUREMENTS

The air-annealed samples are imaged in the tapping mode either with a XE 100 AFM (Park Scientific, Suwon, Korea) or with a Nanoscope Multimode III (Bruker, Billerica MA, USA). AFM cantilevers are type NSC15 (μMash, Sofia, Bulgaria) having a resonance frequency of 325 kHz and a stiffness of 46 N/m. NC-AFM experiments are conducted with a RT-AFM/STM system (Omicron NanoTechnology, Taunusstein, Germany) in a UHV chamber maintaining a base pressure in the low 10^{-10} mbar range. Conducting silicon cantilevers (NanoWorld AG, Neuchatel, Switzerland) are used, which have either a frequency of 70 kHz (type:

PPP-QFMR) or between 280 and 320 kHz (type: PPP-QNCHR). The instrument is operated in the frequency modulation mode with a peak-to-peak oscillation amplitude of around 10 nm where frequency demodulation is accomplished by an EasyPLL demodulator (NanoSurf, Liestal, Switzerland). All images shown here represented the surface topography as the tip-sample interaction strength is kept constant during imaging by a feedback loop adjusting the tip-sample distance accordingly. Simultaneously to NC-AFM measurements, Kelvin probe force microscopy (KPFM) is performed in the frequency modulation mode [3], where a DC and an AC voltage (U_{AC} between 0.5 and 1.0 V peak-to-peak amplitude) with a frequency of $f_{\mathrm{AC}} = 470$ Hz are applied between tip and surface. On insulating surfaces, the distribution of charges and dipoles on the surface is measured [4, 5]. All topography NC-AFM images shown in this work are acquired with the KPFM compensation being active what is important for extracting reliable height information from topography images [6].

XPS MEASUREMENTS

X-Ray photoelectron spectroscopy is accomplished with Mg K_α radiation (1253.6 eV) delivered by an unmonochromatized X-ray source (PSP Vacuum Technology, Macclesfield, UK) operated at 200 W. Photoelectron spectra are recorded with a hemispherical analyzer Resolve 120 with 5-channel detection (PSP Vacuum Technology, Macclesfield, UK), using a pass energy of 50 eV for the survey spectrum and 20 eV for detail spectra. All XPS spectra are taken with the sample kept at room temperature.

To determine the contribution of contaminants like hydroxyls and carbon as well as the degree of oxidation, the C1s, Ce3d and O1s peaks of the XPS spectra taken after different film preparation steps are analyzed. After removing the inelastic background, each spectrum is normalized by its own integrated intensity to remove experimental variations due to slight changes in the sample-source distance that could vary from experiment to experiment.

The C1s spectrum (Fig. 3(c)) is normalized to the emission in the entire Ce3d area. The Ce3d multiplet (Fig. 3(a)) is fitted using 3 doublets representing the Ce^{4+} state (shaded area in red) and two doublets for Ce^{3+}(shaded area in blue), as described in Refs. [7, 8]. The relative contribution of the Ce^{3+} doublet is calculated as 100 $*$ (Ce^{3+} / (Ce^{3+}+ Ce^{4+})) providing a measure for the reduction state[9]. Note that some of the fits do not perfectly match the experimental spectra, which is due to the background subtraction that was difficult to accomplish. Furthermore, we have decided to keep the multiplet parameters (shape, rel. intensities, positions, widths, etc.) as given by Refs. [7, 8]. We estimate the error to be around $\pm5\%$ on the Ce^{3+} concentration.

The O1s spectrum (Fig. 3(b)) is normalized to the corresponding Ce3d integrated intensities, which we assume to be constant over the thermal treatments - the O1s normalized intensity should reflect the changes in the oxygen surface stoichiometry with respect to the cerium concentration. The O1s spectrum is fitted with four Gaussian functions centered at 530.2 eV (peak 1), 531.4 eV (peak 2), 532.6 eV (peak 3) and 533.3 eV (peak 4). We assign peaks 1 and 2 to oxygen bound to Ce^{4+} (O-Ce^{4+}) and Ce^{3+} (O-Ce^{3+}), respectively, representing structural oxygen of CeO_2 and Ce_2O_3 [10, 11]. Peak 3 centered at 532.6 eV is assigned to hydroxyls or H_2O molecules bound to Ce^{3+} [12, 13] whereas peak 4 is assigned to SiO_2 [14].

[1] R. Olbrich, H. H. Pieper, R. Oelke, H. Wilkens, J. Wollschläger, M. H. Zoellner, T. Schroeder, and M. Reichling, Appl. Phys. Lett., **104**, 081910 (2014)

[2] C. Barth, C. Claeys, and C. R. Henry, Rev. Sci. Instr., **76**, 083907 (2005)

[3] S. Kitamura and M. Iwatsuki, Appl. Phys. Lett., **72**, 3154 (1998)

[4] C. Barth and C. R. Henry, Nanotechnology, **17**, S155 (2006)

[5] C. Barth and C. R. Henry, Phys. Rev. Lett., **98**, 136804 (2007)

[6] S. Sadewasser and M. C. Lux-Steiner, Phys. Rev. Lett., **91**, 266101 (2003)

[7] T. Skála, F. Sutara, M. Skoda, K. C. Prince, and V. Matolín, J. Phys.: Condens. Matter, **21**, 055005 (2008)

[8] T. Skála, F. Sutara, K. C. Prince, and V. Matolín, Journal of Electron Spectroscopy and Related Phenomena, **169**, 20 (2009)

[9] P. Luches, F. Pagliuca, and S. Valeri, Phys. Chem. Chem. Phys., **16**, 18848 (2014)

[10] D. R. Mullins, S. H. Overbury, and D. R. Huntley, Surf. Sci., **409**, 307 (1998)

[11] T. Hasegawa, S. M. F. Shahed, Y. Sainoo, A. Beniya, N. Isomura, Y. Watanabe, and T. Komeda, J. Chem. Phys., **140**, 044711 (2014)

[12] L. Kundakovic, D. R. Mullins, and S. H. Overbury, Surf. Sci., **457**, 51 (2000)

[13] M. A. Henderson, C. L. Perkins, M. H. Engelhard, S. Thevuthasan, and C. H. F. Peden, Surf. Sci., **526**, 1 (2003)

[14] E. J. Preisler, O. J. Marsh, R. A. Beach, and T. C. McGill, J. Vac. Sci. Technol. B, **19**, 1611 (2001)
